# Predictors of early progression of surgically treated atypical meningiomas

**DOI:** 10.1007/s00701-018-3593-x

**Published:** 2018-06-30

**Authors:** Karol P. Budohoski, James Clerkin, Christopher P. Millward, Philip J. O’Halloran, Mueez Waqar, Seamus Looby, Adam M. H. Young, Mathew R. Guilfoyle, Diana Fitzroll, Abel Devadass, Kieren Allinson, Michael Farrell, Mohsen Javadpour, Michael D. Jenkinson, Thomas Santarius, Ramez W. Kirollos

**Affiliations:** 1 Division of Neurosurgery, Cambridge Biomedical Campus, Addenbrooke’s Hospital, University of Cambridge, Box 167, Cambridge, CB2 0QQ UK; 2 Department of Neurosurgery, Beaumont Hospital, Dublin, Ireland; 3 Department of Neurosurgery, The Walton Centre, Liverpool, UK; 4 Institute of Translational Medicine, University of Liverpool, Liverpool, UK; 5 Department of Neuropathology, Addenbrooke’s Hospital, University of Cambridge, Cambridge, UK; 6 Department of Pathology, Beaumont Hospital, Dublin, Ireland

**Keywords:** Atypical meningioma, Early recurrence, Early progression, Predictors of recurrence

## Abstract

**Background:**

Clinical behaviour of atypical meningiomas is not uniform. While, as a group, they exhibit a high recurrence rate, some pursue a more benign course, whereas others progress early. We aim to investigate the imaging and pathological factors that predict risk of early tumour progression and to determine whether early progression is related to outcome.

**Methods:**

Adult patients with WHO grade II meningioma treated in three regional referral centres between 2007 and 2014 were included. MRI and pathology characteristics were assessed. Gross total resection (GTR) was defined as Simpson 1–3. Recurrence was classified into early and late (≤ 24 vs. > 24 months).

**Results:**

Among the 220 cases, 37 (16.8%) patients progressed within 24 months of operation. Independent predictors of early progression were subtotal resection (STR) (*p* = 0.005), parafalcine/parasagittal location (*p* = 0.015), peritumoural oedema (*p* = 0.027) and mitotic index (MI) > 7 (*p* = 0.007). Adjuvant radiotherapy was negatively associated with early recurrence (*p* = 0.046). Thirty-two per cent of patients with residual tumour and 26% after GTR received adjuvant radiotherapy. There was a significantly lower proportion of favourable outcomes at last follow-up (mRS 0–1) in patients with early recurrence (*p* = 0.001).

**Conclusions:**

Atypical meningiomas are a heterogeneous group of tumours with 16.8% patients having recurrence within 24 months of surgery. Residual tumour, parafalcine/parasagittal location, peritumoural oedema and a MI > 7 were all independently associated with early recurrence. As administration of adjuvant radiotherapy was not protocolised in this cohort, any conclusions about benefits of irradiation of WHO grade II meningiomas should be viewed with caution. Patients with early recurrence had worse neurological outcome. While histological and imaging characteristics provide some prognostic value, further molecular characterisation of atypical meningiomas is warranted to aid clinical decision making.

## Introduction

Intracranial meningiomas constitute 35% of all primary brain tumours and are generally considered benign. [1] Nevertheless, atypical meningiomas, which account for 20–35% of all meningiomas, have recurrence rates up to 50% and 10-year survival less than 80%. [2–4]

There are numerous histological subtypes of meningioma; however, the WHO classification is typically used to determine the biological behaviour, i.e. the risk of recurrence or progression. Since the changes in diagnostic criteria introduced in 2000, there has been a significant increase in the reported incidence of WHO grade II tumours from approximately 5% before 2000 to 30% of all meningiomas in more recent series. [5–7] The median time to progression of atypical meningiomas is approximately 24 months, [8–10] and they remain a heterogeneous group of tumours with reports of tumour progression within 1 year of operation despite gross total resection (GTR). [11] Due to the heterogeneity, there is no uniform treatment paradigm currently being used to treat atypical meningiomas. The role of adjuvant radiotherapy as well as the frequency and length of follow-up remain to be determined. [12] A number of studies have aimed to identify the clinical and histological characteristics which can be used to predict recurrence and justify more aggressive treatment. [6, 10, 13–27] Subtotal resection, [10, 15, 16, 20] brain invasion, [16, 23, 25, 26, 28] high mitotic index (MI), [7, 10, 26, 28] high proliferation index (MIB-1/Ki-67), [15, 17] absence of EGFR receptor, [24] bone involvement [19, 23] and progression form WHO grade I [10, 29, 30] have all been implicated in prognosis.

Nevertheless, there remains a paucity of data concerning the exact timing of progression and its implication for prognosis. The aim of this study is to identify routinely available imaging and histological characteristics that may be associated with early recurrence/progression of WHO grade II meningiomas.

## Methods

Retrospective analysis of all meningiomas from the histopathological records of three regional neurosurgery units. All patients diagnosed as WHO grade II meningioma were included. Only patients diagnosed before 2014 were included to allow minimum 2-year follow-up. Each Institutional Review Board approved this study.

Early aggressive behaviour was defined as radiological recurrence or progression (see below for definitions) within the first 2 years after definitive treatment with surgery (with or without adjuvant radiotherapy).

Clinical and patient characteristic used in the analysis included the following: age at diagnosis, extent of resection, the use of adjuvant radiotherapy, recurrence of tumour on follow-up imaging, time to recurrence, number of surgeries and outcome at last follow-up. Extent of resection was determined based on post-operative MRI (median time from surgery to imaging 23 days) and/or intraoperative findings. If postoperative imaging and intraoperative findings were in disagreement, the modality that demonstrated residual was favoured. Subtotal resection (STR) was defined as a persistent area of contrast uptake within part of the volume of the original tumour on post-operative MRI scan or when operative report stated that residual tumour was left, i.e. Simpson grades 4 and 5. Gross total resection (GTR) was defined as Simpson 1–3. Recurrence was defined as presence of tumour where there was no tumour on post-operative MRI. Progression of tumour was defined as any detected increase in size of residual tumour documented on follow-up MRI. Adjuvant radiotherapy was defined as radiotherapy administered to the tumour bed within 6 months of surgery to prevent, rather than treat recurrence/progression. We did not stratify patients depending on whether stereotactic radiosurgery or fractionated radiotherapy was performed.

**Fig. 1:**
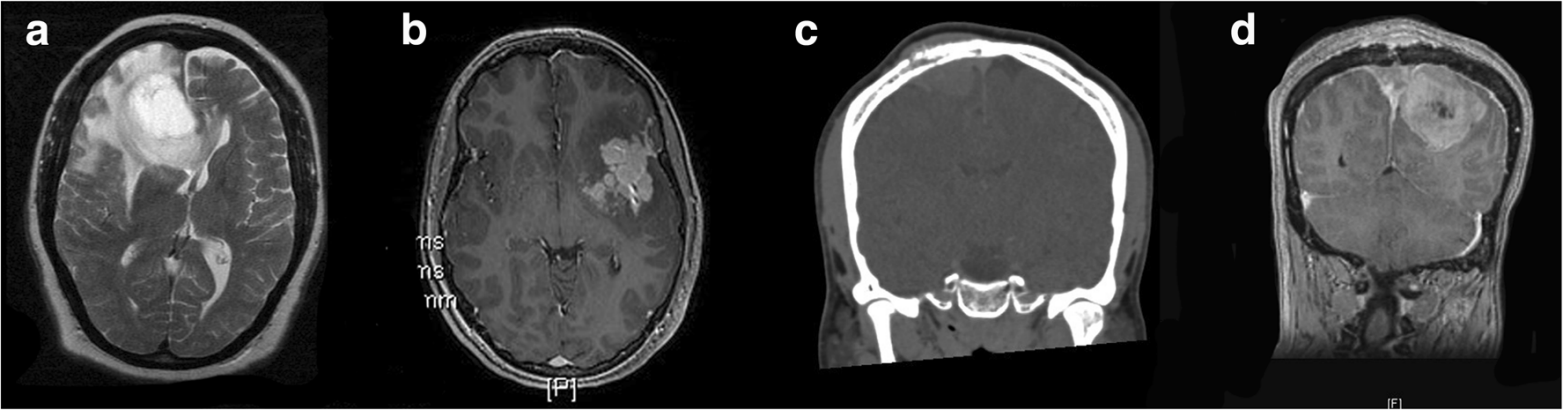
Examples of radiological characteristics used in the study. **a** Peritumoural oedema manifested as T2 hyperintensity immediately surrounding the tumour with mass effect. **b** Irregular margins with ‘mushrooming’ and nodules appearing as if detached from main mass of tumour. **c** Bone involvement in a parasagittal meningioma. **d** Sinus involvement manifest with tumour clearly present in the cavity of the superior sagittal sinus

Imaging characteristics included the following: location of tumour, involvement of dural sinus, bone erosion, irregularity of margins and presence of peritumoural oedema on pre-operative imaging (Fig. 1). Location of tumour was divided into the following: convexity, parafalcine/parasagittal and skull base. Sinus and bone involvement was determined based on the pre-operative imaging, surgical findings and/or pathology reports. Irregularity of margins was determined on pre-operative contrast-enhanced T1 MRI scan and was defined as margins displaying at least one area of irregularity, daughter nodule or area of mushrooming. [31] Peritumoural oedema was determined on pre-operative MRI scans and was defined as T2 hyperintensity seen within the brain surrounding the contrast enhancing tumour (after excluding other possible causes, e.g. known infarct, multiple sclerosis etc.).

Pathology characteristics included the following: brain invasion, atypia, necrosis, MI (reported as number of mitotic figures seen per 10 high power fields [HPF]) and MIB1 count. All pathology reports underwent central review to confirm that diagnosis was in keeping with 2016 WHO criteria.

All patients had a minimum of 2 years of follow-up. Outcome was categorised using the modified Rankin Scale (mRS) at the last available clinic appointment. For statistical analysis, patients were dichotomised into those with favourable (mRS 0–1) and unfavourable (mRS ≥ 2) outcomes.

## Statistical analysis

The median time to recurrence/progression was determined. Patients in whom recurrence/progression was seen before the median time (as defined for the whole cohort) were included in the ‘early recurrence/progression’. Patients in whom recurrence/progression was seen after the median time (as defined for the whole cohort), or those did not progress until last follow-up, were labelled as ‘others’.

Receiver operator characteristic (ROC) curve data was used to dichotomise continuous variables such as MI and MIB1. MI was dichotomised at > 7/10 high power fields (HPF) while MIB1 was dichotomised at > 15%.

Kaplan-Meier curves with Mantel Cox test were used to assess relationships between patient/clinical, radiological and pathology factors and progression-free survival. Multivariate logistic regression was used to determine factors independently associated with early recurrence/progression. Variables found significant on univariate analysis were included in the multivariate model. Sensitivity analysis for factors found to be independently associated with early recurrence was performed. Chi square was used to determine whether early recurrence/progression is associated with worse outcomes in patients with atypical meningioma.

Statistical analysis was performed using SPSS software (SPSS, IBM, USA).

## Results

We identified 220 patients diagnosed with WHO II atypical meningiomas (Table 1). Data for extent of resection was available for 205 patients. GTR was achieved in 143 patients. Mean (overall survival has not reached a median, hence mean reported) overall survival (OS) for the whole cohort was 159 months while median progression-free survival (PFS) was 68 months. Five- and 10-year OS was 87 and 69%, and PFR was 59 and 19%.

**Table 1:** Baseline characteristics

Factor		*n*=
*N*		220
Female (%)		122 (56%)
Age, median (IQR)		61 (50–68)
Recurrence	Overall (%)	71 (32%)
	Recurrence within 1 year (%)	18 (8%)
	Recurrence within 2 years (%)	37 (17%)
Months to recurrence, median (IQR)		24 (12–43)
Location	Convexity (%)	103 (47%)
	Parafalcine (%)	38 (17%)
	Skull base (%)	50 (23%)
	Intraventricular (%)	5 (2%)
	Sinus involvement (%)	26 (12%)
STR (%)		62 (28)
Radiotherapy	Adjuvant (%)	57 (26%)
	For recurrence (%)	34 (16%)
mRS, median (IQR)		1 (1–3)
mRS ≤ 1		73%
mRS ≤ 2		83%

Recurrence within 1 year and within 2 years refers to a recurrence up to and including 12 and 24 months post-operatively, respectively

*IQR* interquartile range, *mRS* modified Rankin score, *STR* subtotal resection

Seventy-one patients (32%) had recurrence or progression. Of patients that recurred, the median time to recurrence was 24 months (IQR 12–43). Patients who experienced tumour recurrence within 24 months after treatment comprised the ‘early recurrence/progression’ group. Table 2 demonstrates the numbers of patients with early and any recurrence depending on extent of resection stratified by location. Briefly, of patients with GTR 12% had early recurrence, 27% had any recurrence at last follow-up. On the other hand, of the patients with STR, 32% had early recurrence, 50% had any recurrence at last follow-up. On univariate analysis, extent of resection was significantly related to the rates of early (*p* = 0.005) and any recurrence (*p* = 0.002). However, when specific locations were examined, only early recurrence of tumours located at the convexity, but not tumours in the parafalcine/parasagittal location, skull base, nor those involving the sinuses, seemed to be significantly higher in the STR group.

**Table 2:** Differences in early and any recurrence stratified by location, extent of resection and the use of adjuvant radiotherapy

	*n*	Early recurrence, *n* (%)		Any recurrence, *n* (%)	
All, GTR	143	17 (12)	*p* = 0.005	39 (27)	*p* = 0.002
All, STR	62	20 (32)		31 (50)	
Convexity, GTR	79	5 (6)	*p* = 0.001	17 (22)	*p* = 0.01
Convexity, STR	22	7 (32)		11 (50)	
Parafalcine/parasagittal, GTR	18	8 (44)	*p* = 0.64	9 (50)	*p* = 0.44
Parafalcine/parasagittal, STR	19	7 (37)		11 (58)	
Skull base, GTR	26	4 (15)	*p* = 0.077	9 (35)	*p* = 0.14
Skull base, STR	18	7 (39)		10 (56)	
Sinus involvement, GTR	5	3 (60)	*p* = 0.12	4 (80)	*p* = 0.27
Sinus involvement, STR	21	5 (24)		11 (52)	
Adjuvant XRT	57	7 (12)	*p* = 0.049	14 (26)	*p* = 0.09
No XRT	140	28 (20)		50 (36)	
Adjuvant XRT, GTR	35	3 (9)	*p* = 0.22	9 (26)	*p* = 0.61
Adjuvant XRT, STR	20	4 (20)		5 (25)	

*STR* subtotal resection, *XRT* adjuvant radiotherapy; any recurrence—defined as recurrence within the period of follow-up

Fifty-seven patients received adjuvant radiotherapy. Of those, 35 received prophylactic adjuvant radiotherapy despite GTR, while 22 underwent radiotherapy for residual. A further 34 patients had radiotherapy for recurrence. Table 2 describes the numbers of patients with recurrence stratified by the use of adjuvant radiotherapy (patients who underwent radiotherapy for recurrence are not included in the table).

**Fig. 2:**
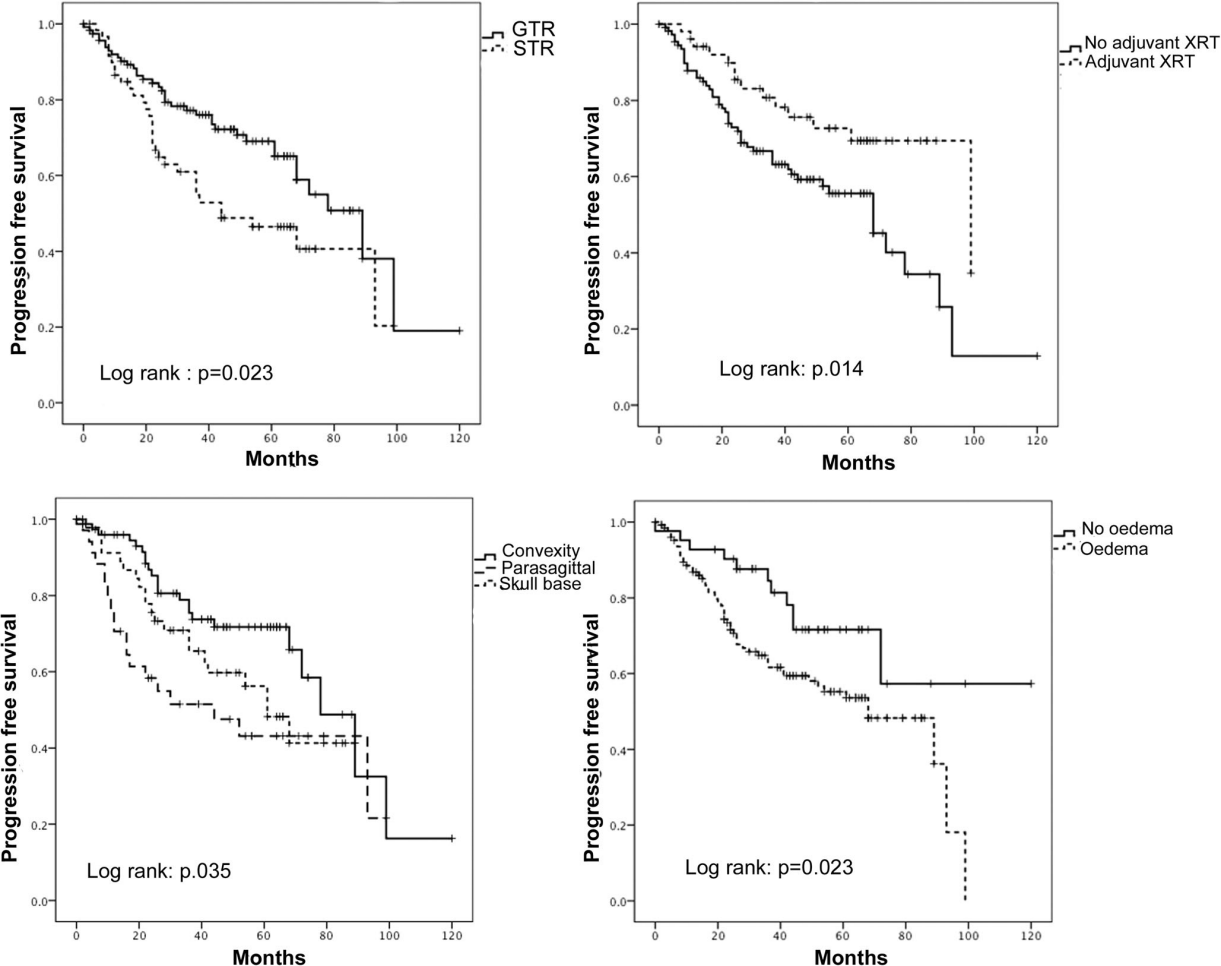
Kaplan-Meier plots demonstrating a significant association between extent of resection; the use of adjuvant XRT; location (divided into convexity, parafalcine/parasagittal and skull base); peritumoural oedema and progression-free survival for patients with atypical meningiomas. Log rank test for significance used to determine statistical significance

**Fig. 3:**
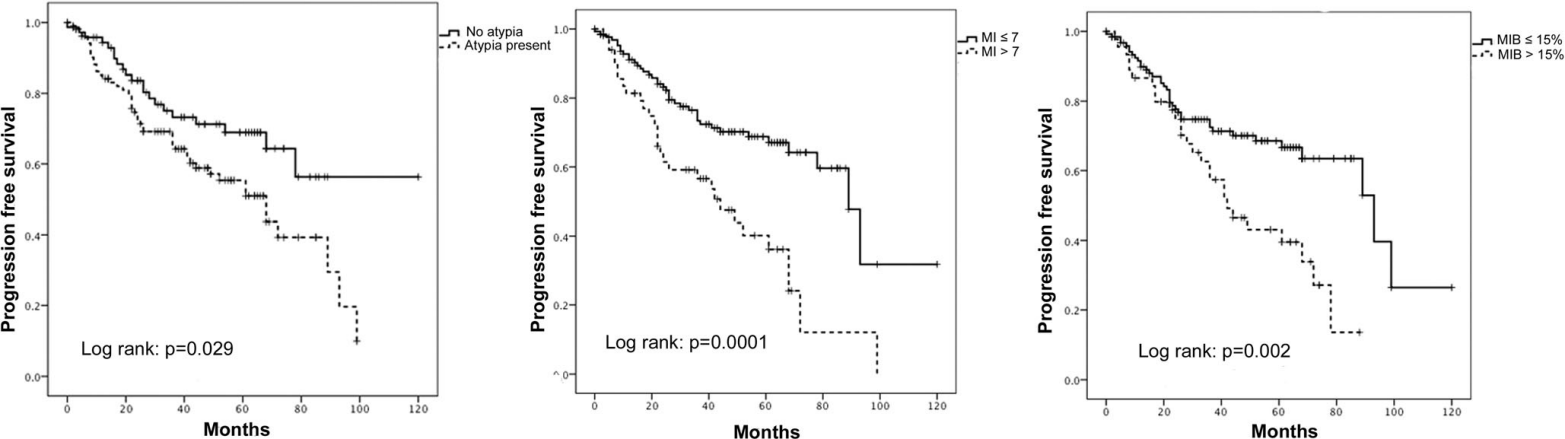
Kaplan-Meier plots demonstrating a significant association between presence of atypia; MI; MIB1 count and progression-free survival. MI has been dichotomised to MI ≤ 7/10 HPF and MI > 7/10 HPF and MIB1 has been dichotomised to MIB1 ≤ 15% and MIB1 > 15%. Log rank test for significance used to determine statistical significance

Figure 2 demonstrates the Kaplan-Meier plots for progression-free survival stratified by extent of resection, the use of adjuvant radiotherapy, location of tumour and presence of peritumoural oedema. Figure 3 demonstrates the Kaplan-Meier plots for progression-free survival stratified by pathological characteristics of atypia, MI and MIB1. On univariate analysis, all factors apart from necrosis, presence of irregular margins and brain invasion were significantly associated with progression-free survival.

Independent predictors of early recurrence/progression using multivariate logistic regression were STR (*p* = 0.005), parafalcine/parasagittal location (*p* = 0.015), peritumoural oedema on pre-operative MRI (*p* = 0.027) and MI > 7 (*p* = 0.007), while adjuvant radiotherapy was negatively associated with early progression (*p* = 0.046) (Table 3). No other clinical, imaging nor pathological characteristics were found to be independently associated with the risk of early recurrence. Of the 62 patients with STR, 20 (32%) received adjuvant radiotherapy. A further 37 patients received adjuvant radiotherapy after GTR. When logistic regression was repeated including only patients who underwent GTR, the use of adjuvant radiotherapy was no longer negatively associated with early recurrence (*p* = 0.37; OR 0.52 [0.13–2.16]).

**Table 3:** Predictors of recurrence of atypical meningioma—multivariate regression

		OR	95% CI for OR	*p*
STR		3.62	1.48–8.88	*p* = 0.005
Adjuvant XRT		0.38	0.29–0.97	*p* = 0.046
Location	Convexity	0.85	0.29–2.46	*p* = 0.77
	Parafalcine	3.81	1.29–11.22	*p* = 0.015
	Skull base	2.95	0.91–9.62	*p* = 0.07
Imaging	Oedema	4.62	1.19–17.90	*p* = 0.027
Pathology	Atypia	1.14	0.39–3.38	*p* = 0.81
	MI > 7/10 HPF	4.27	1.40–12.19	*p* = 0.007

*CI* confidence interval, *HPF* high power field, *MI* mitotic index, *OR* odds ratio, *STR* subtotal resection, *XRT* radiotherapy

The presence of oedema on pre-operative MRI had 92% sensitivity and 30% specificity for predicting 24-month recurrence. The sensitivity and specificity of MI > 7/10 HPF were more balanced, i.e. 71 and 75%, respectively. STR had a sensitivity and specificity for predicting 24-month recurrence of 54 and 75%, respectively.

**Fig. 4:**
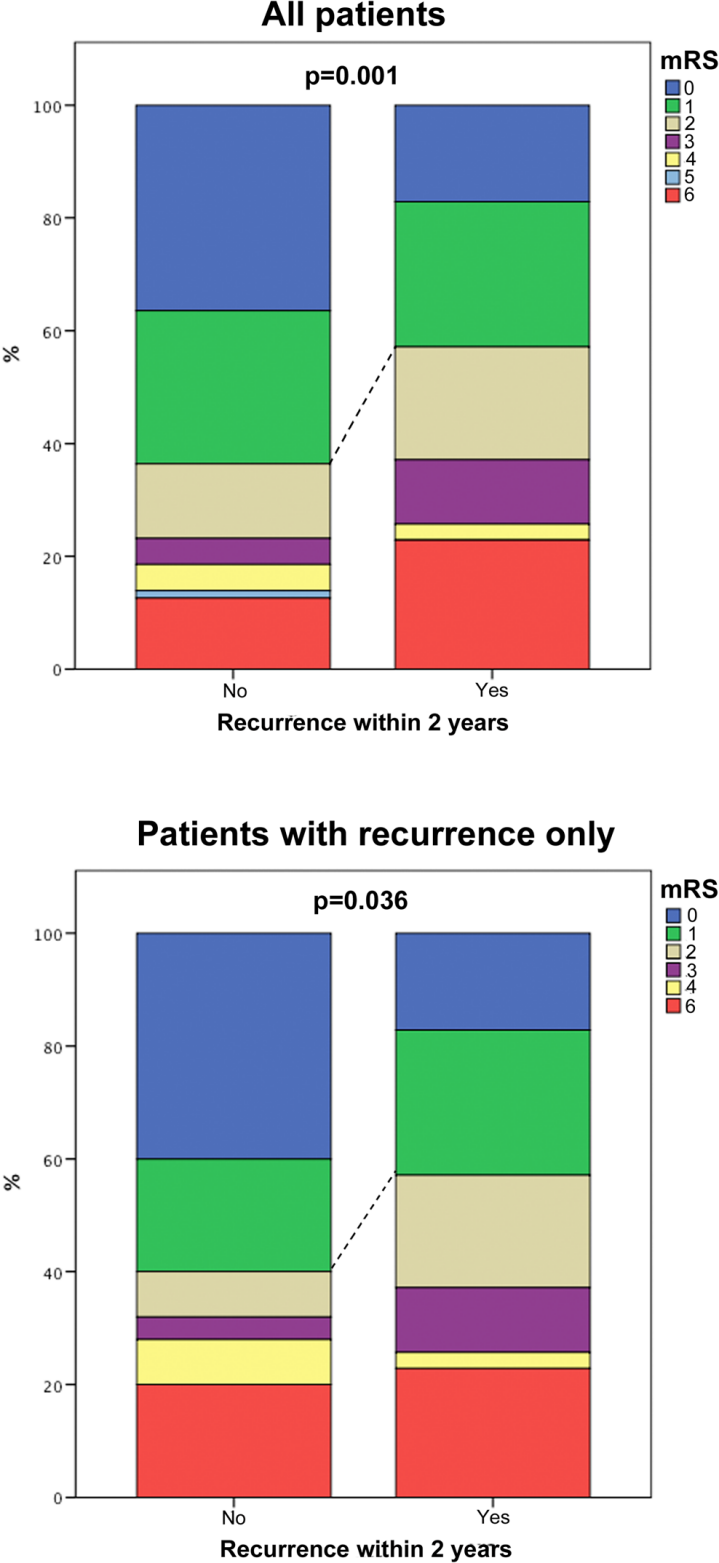
Bar chart demonstrating the difference in clinical outcomes between the ‘early progression/recurrence’ groups. All others (*top graph*); below the same analysis is repeated excluding patient who never had a recurrence (*bottom graph*). Dashed line depicts differences in number of patients with favourable outcomes defined as mRS 0–1 at last follow-up. mRS-modified Rankin Scale

mRS scores were obtained at a median of 54 months post-surgery. There was a significantly lower proportion of patients with favourable outcomes at last follow-up (defined as mRS 0–1) among patients with early recurrence/progression versus others (Fig. 4; *p* = 0.001). Furthermore, this difference remained significant when patients without recurrence were excluded from the analysis (Fig. 4; *p* = 0.036).

## Discussion

In this study, we analysed the usefulness of the routinely available clinical, radiological and pathological characteristics in predicting early disease recurrence and/or progression within 24 months of surgical treatment, in patients with WHO grade II meningioma. In our series, subtotal resection, parafalcine/parasagittal location, peritumoural oedema visible on pre-operative imaging and a mitotic index > 7/10 HPF were independently associated with early recurrence. Furthermore, in this cohort of patients, the use of adjuvant radiotherapy was associated with a reduced rate of early recurrence within 24 months. Importantly, we also found that patients who exhibit early recurrence of WHO II meningioma have a less favourable functional outcome, both when compared with the overall population of patients with WHO II meningiomas as well as when compared with patients who had recurrence later than 24 months after treatment.

Atypical meningioma is a heterogeneous group of tumours. There have been a number of reports looking into factors associated with progression-free survival with multiple factors being implicated. Location, [10, 32] extent of resection, [15, 17, 20, 33–35] presence of atypia, [36] brain invasion, [23, 26, 37, 38] high MI, [26, 37–39] high MIB1 labelling, [15, 17, 33, 39] bone involvement, [19, 23, 37] use of adjuvant radiotherapy [9, 40–42] and secondary progression from WHO I tumour. [30] However, others have shown that none of the above factors influence the recurrence rate or time to recurrence of atypical meningioma. [43]

Extent of resection is a known predictor of the risk of recurrence of meningiomas. [15, 17, 20, 33–35] Our study shows that this is relevant to WHO grade II meningiomas, such that STR was independently associated with early recurrence/progression within 24 months. In our study, 54% of meningiomas with early recurrence/progression had a known residual. We have pragmatically used GTR vs. STR to define extent of resection, as we recognise that in our retrospective series involving multiple surgeons, it was impossible to differentiate with sufficient rigour patients who underwent Simpson 1 vs. Simpson 2 vs. Simpson 3 resections. Consequently, our data do not provide information on the benefits of different Simpson grade resections separately. Furthermore, colinearities, undoubtedly, exist between the extent of resection and use of adjuvant radiotherapy. However, radiotherapy in this group of patients was not used in a systematic way, and only one third of patients with residual tumour received adjuvant radiotherapy while the other two thirds did not.

Apart from subtotal resection, we identified only two radiological and one histological characteristics associated with early aggressive behaviour and recurrence/progression within 24 months after treatment. Only parafalcine/parasagittal location and peritumoural oedema seen on preoperative MRI were independently related to early recurrence. Some reports have suggested that there may be a relationship between location and recurrence rate of meningiomas. [10, 32] Whether there is a certain biological makeup of tumours related to their location which predisposes to recurrence is unknown. [44] It is likely that overall higher recurrence rates observed in parafalcine/parasagittal location is representative of only being able to achieve STR in this location with residual tumour invading the superior sagittal sinus. Fifty per cent of patients with tumours in the parafalcine/parasagittal location were known to have a residual visible on post-operative imaging. Nevertheless, univariate analysis demonstrated that early recurrence rates were close to 40% regardless of whether GTR or STR was achieved. Although we do not have data on the genetic makeup of the tumours in this location, nor do we have more detailed descriptions of extent of resection than post-operative imaging and operative reports to make definitive statements, we believe that both the univariate and multivariate analyses confirm higher early recurrence rates in parafalcine/parasagittal meningiomas irrespectively of the extent of resection. Furthermore, while Simpson grade 1 resection would be desirable if recurrence was the only consideration, in real life, there are many other important considerations, not least widely published morbidity related to radical resection of meningiomas invading venous sinuses. [45, 46] Indeed some authors propose that use of stereotactic radiosurgery following incomplete resection of parasagittal meningiomas reduces recurrence rates to those seen with Simpson grade 1 resections. [47] While we have notinvestgated this directly our data does not support pursuing radical resection in parafalcine/parasagittal meningiomas at the expense of morbidity.

In this cohort, brain invasion was not found to be associated with early tumour recurrence. It is widely accepted that diagnosis of brain invasion using operative samples is difficult as frequently brain tissue is not included in the sample. [48] We were not able to ascertain whether the samples provided for central review were representative for assessing brain invasions and this constitutes a limitation of this study. We have, however, re-analysed our data including only samples where brain tissue was present and a definitive statement about brain invasion could have been made. However, this analysis did not change the result and brain invasion was not found to be independently associated with early recurrence on multivariate analysis in this limited sample.

Our understanding of the pathophysiology of peritumoural oedema associated with meningiomas remains incomplete. Previous studies have implicated size, [49] growth rate, [50] leptomeningeal invasion, development of pial blood supply, [51, 52] as well as specific histological types [51, 53] with development of peritumoural oedema. In our series, the presence of peritumoural oedema was significantly associated with early aggressive behaviour and recurrence at 24 months. Oedema had a 92 and 30% sensitivity and specificity, respectively, suggesting that it may be used as a guide to determine frequency of surveillance but may not be specific enough to warrant routine delivery of adjuvant radiotherapy.

The histological diagnosis of atypical meningioma is based on the presence of the following: high MI 4–19/10 HPF, specific features of atypia (hypercellularity, prominent nucleoli, diffuse growth pattern, necrosis and small cell change) or brain invasion. Of those routinely available histological parameters (and MIB1 labelling), only a MI > 7/10 HPF was independently associated with early progression in our study. Indeed, a high MI has been previously reported to be related to overall recurrence of atypical meningioma, but not early recurrence. [17, 26, 38, 39] As atypical meningiomas have a narrowly defined range of MI, the value of this parameter is likely diminished. For this reason, most studies do not give a threshold MI related to recurrence, but treat the presence of high MI (i.e. > 4/10 HPF) as a factor. In this study, based on a ROC curve analysis, 7 mitoses/10 HPF were determined as the threshold value in our study. This is in keeping with a report by Sun et al. [26] who also found MI > 7/10 HPF related to a higher rate of recurrence in completely resected atypical meningiomas (particularly in the absence of brain invasion). MI > 7/10 HPF had a sensitivity of 71% and specificity of 75% for predicting 24-month recurrence. No other histological characteristic was associated with early recurrence.

The role of adjuvant radiotherapy in the management of atypical meningioma is not fully defined. [54] Similarly to our study, literature typically reports results of radiotherapy independently of the extent of resection as well as tumour location. While a relationship between reduced rates of recurrence and the use of adjuvant radiotherapy following surgery for atypical meningiomas has been previously shown, [9, 40–42] there have been individual reports raising concern that, in fact, radiotherapy may transform meningiomas into more aggressive or anaplastic types. [55, 56] Indeed, in a series of 610 meningiomas, a 2.2% rate of malignant transformation at a median of 4.9 years after SRS has been reported. [56] In our series, 56 patients underwent adjuvant radiotherapy; however, only one third of those patients had residual tumour, while the other two thirds were prophylactically irradiated based on patient and clinician preference on the premise of preventing future recurrence. In our study, adjuvant radiotherapy was independently associated with a reduced risk of early recurrence/progression when all patients were analysed. However, this was not the case when only patients with GTR were analysed suggesting that there may be less benefit in prophylactic adjuvant radiotherapy. Due to the variable clinical indications for adjuvant radiotherapy and the inherent bias this introduces, we cannot conclude that radiotherapy should be used for all patients. Two large, multicentre international randomised controlled trials are in progress and will ultimately address the role of early adjuvant radiotherapy for atypical meningioma. [12, 57]

Whilst our data do not provide definitive answers, we can postulate that early progression/recurrence of atypical meningioma may be related as much to the aggressiveness of treatment as well as biological makeup of the specific tumours. While some characteristics routinely available in clinical practice can aid in prognostication and are very important for day-to-day treatment decisions, this study further demonstrates the heterogeneity of atypical meningiomas and the need for developing risk stratification tools, which go beyond the WHO grading system. A number of mutations as well as DNA methylation profiles have all been shown to be linked with the risk of recurrence in meningioma. [58, 59] Addition of molecular markers has the potential to significantly improve not only understanding of the biology of meningioma, but refine prognostic and treatment stratification as well as development of more targeted treatment modalities. Importantly, this study has demonstrated that early recurrence/progression of atypical meningioma was significantly related to neurological outcomes. Therefore, identification of clinical, biological and molecular predictors of recurrence is crucial to rationally stratify management decisions.

Our study has several limitations, which need to be acknowledged. Firstly as a retrospective analysis, we relied on clinical documentation, particularly related to extent of resection. While we have taken all possible measures to minimise this bias, we are aware that inaccuracies could have been introduced. Overall survival in patients with meningiomas is difficult to ascertain, as long observation periods are required. The available survival data only allowed an analysis of all cause mortality, rather than tumour-specific mortality. Furthermore, we did not have age-matched life expectancy data for comparison. Whilst there was a trend towards better tumour control in those treated with radiotherapy, this needs to be further evaluated and two international phase III trials are ongoing (NRG BN-003 (http://clinicaltrials.gov/ct2/show/NCT03180268) and the ROAM trial (http://roam-trial.org.uk)). Finally, while central pathology review was possible to determine the MIB1 and MI, we were not able to review all pathology slides to comprehensively assess brain invasion and instead we had to rely on available pathology reports.

## Conclusions

We have identified a specific group of tumours within this cohort of atypical meningioma characterised by early aggressive behaviour and recurrence within 24 months after initial surgical treatment. We have demonstrated that such early recurrence was related to poor neurological outcome.

Parafalcine/parasagittal location, peritumoural oedema on pre-operative MRI scan as well as a MI > 7/10 HPF  were positively associated, while the use of adjuvant radiotherapy was negatively associated with the risk of early recurrence. While the radiological and pathological characteristics were found to be sensitive, they were not specific enough to automatically mandate adjuvant treatment.

We have demonstrated that adjuvant radiotherapy was associated with a reduced risk of early recurrence. Nevertheless, limited sample size and inconsistent use of radiotherapy in this cohort prevent us from making a definitive statement. The role of adjuvant radiotherapy remains to be determined in prospective studies.

Overall, the routinely available radiological and histological parameters are insufficient to accurately predict behaviour and stratify management of patients with this heterogeneous group of tumours. It is likely that molecular markers, like in other neoplastic diseases, will fill this void and future research should be focused in this direction.
